# Divisive and non-monotonic gain control in open-loop neural circuits

**DOI:** 10.1186/1471-2202-14-S1-P248

**Published:** 2013-07-08

**Authors:** Jorge F Mejias, Alexandre Payeur, Erik Selin, Leonard Maler, Andre Longin

**Affiliations:** 1 Department of Physics, University of Ottawa, Ottawa, ON, K1N6N5, Canada; 2 Department of Cellular and Molecular Medicine, University of Ottawa, Ottawa, ON, K1H8M5, Canada; 3 Centre for Neural Dynamics, University of Ottawa, Ottawa, ON, K1N6N5, Canada

## 

The proper characterization of input-output properties of neurons is a long-standing goal in neuroscience. A common way to study such properties is by means of the so called f-I curve, which measures the output firing rate of a neuron as a response of a given level of constant input current [1]. In many cases, an average slope or *gain *characterizing the response may be obtained. This method is particularly interesting to understand the response properties of neurons to slowly modulated input, although extensions to other situations are also possible [2].

Mechanisms able to modulate the neural input-output properties have been extensively studied in this framework. For instance, a shift in the leak conductance of the neuron can cause subtractive effects in the f-I curve [3]. Mechanisms responsible for other forms of gain control, such as divisive or nonlinear effects, have been more elusive [3,4], and only a number of them are known [4,5].

We present here a computational model of a neural circuit which is able to display these two types of gain control mechanisms (i.e. divisive and nonlinear), in addition to the standard subtractive gain control. We have considered a circuit which models the indirect feedback pathway to the superficial pyramidal (SP) neurons, a cerebellar-like structure found in the electrosensory lateral-line lobe of the weakly electric fish. This fish is able to generate an oscillatory electric field which is used for prey detection and communication. Electroreceptors located in the fish' skin detect the modulations in the electric field caused by stimuli, e.g. other fish, and project to SP neurons and to another subpopulation of pyramidal cells called deep pyramidal (DP) neurons. The population of DP neurons then activates the feedback pathway, which provides a net inhibitory signal to the SP cells.

We have found that the effect of the contribution of the feedback pathway is to divisively reduce the firing rate of the SP neurons, thus acting as a divisive gain control mechanism. This illustrates how divisive gain control may be obtained in open-loop recurrent circuits. In addition, we analyzed the conditions in which the f-I response curve of the SP neurons becomes non-monotonic, thus revealing a novel nonlinear gain control mechanism which agrees with *in vitro *experimental recordings in the electric fish [6].
